# Subcutaneous ureteral bypass explantation in 21 cats (2015-2023): outcomes and owner perceptions

**DOI:** 10.1093/jvimsj/aalag197

**Published:** 2026-09-02

**Authors:** Sophie E Broughton, Anna Frykfors von Hekkel, Matteo Rossanese, Lynda Rutherford, Rebecca F Geddes, Jack S Lawson

**Affiliations:** Clinical Science and Services, Royal Veterinary College, Hertfordshire AL9 7TA, United Kingdom; Comparative Biomedical Sciences, Royal Veterinary College, London NW1 0TU, United Kingdom; Clinical Science and Services, Royal Veterinary College, Hertfordshire AL9 7TA, United Kingdom; Clinical Science and Services, Royal Veterinary College, Hertfordshire AL9 7TA, United Kingdom; Clinical Science and Services, Royal Veterinary College, Hertfordshire AL9 7TA, United Kingdom; Hospital for small animals, University of Edinburgh, Edinburgh EH25 9RG, United Kingdom; Clinical Science and Services, Royal Veterinary College, Hertfordshire AL9 7TA, United Kingdom; Clinical Science and Services, Royal Veterinary College, Hertfordshire AL9 7TA, United Kingdom

**Keywords:** bypass, calcium, feline, nephrolithiasis, ureteral, ureteric obstruction

## Abstract

**Background:**

Subcutaneous ureteral bypass (SUB) can be an effective treatment for ureteral obstruction in cats, but SUB explantation is little documented.

**Hypothesis/Objectives:**

Describe reasons for SUB explantation, outcomes, and owner experiences. We hypothesized that explantation would be effective in managing common SUB-related complications.

**Animals:**

Twenty-one cats (31 SUBs) that underwent 29 explantations.

**Methods:**

Retrospective case-series of cats undergoing explantation between 2015 and 2023 with medical record review and 20 owners emailed for follow-up. Results are median (range).

**Results:**

Explantation was performed 1.8 years (0.2-4.1 years) after placement because of positive urine culture (PUC; 15/21), SUB obstruction (8/21), sterile cystitis (4/21), and suspected SUB failure (2/21). Twenty-six out of 31 ureters had documented patency. Seventeen cats had all implants removed (9 unilateral, 8 bilateral). Two underwent unilateral explantation and ipsilateral ureteronephrectomy. Two with bilateral SUBs underwent unilateral explantation. Follow-up after explantation was 596 days (3-2310 days). Four cats had ureteral re-obstruction (1 after partial obstruction at explantation), 10 did not re-obstruct and the remainder were undetermined. Seven cats died at 614 days (405-2200 days), 11 were lost to follow-up at 135 days (19-1774 days) after explantation, and 3 remained alive. Four deaths were renal related (2/4 re-obstructions). Nine of 15 cats explanted due to PUC had a negative postoperative urine culture. Three of 15 cats had clinical signs related to PUC postoperatively. Questionnaire response was 9 of 20, and 8 of 9 owners would consider explantation if in the same situation again.

**Conclusions and clinical importance:**

For SUB cats with complications, explantation is a viable option. A small proportion of cats had clinical signs of PUC, or ureteral re-obstruction postoperatively.

## Introduction

Ureteral obstruction is a common cause of acute kidney injury in cats, accounting for approximately 17%-24% of cats where a definitive diagnosis is reached.^1^^,^^2^ Ureterolithiasis is the most frequent cause of ureteral obstruction in cats.^3^ Medical management of obstructive ureterolithiasis in cats is associated with a poor success rate.^4^ Ureterotomy and ureteral stenting are associated with high complication rates,^5–7^ although recent literature suggests more promising outcomes with careful case selection.^8–10^ The ACVIM consensus statement recommends subcutaneous ureteral bypass (SUB) placement or ureteral stenting for obstructive ureterolithiasis in cats,^11^ with SUB placement being the surgical treatment of choice at the authors’ institution for most ureteral obstructions in cats. However, although median survival time for cats undergoing SUB placement is between 274 and >800 days,^3^^,^^12^^,^^13^ long-term complications are common, including implant mineralization and obstruction (21.05%-29.4%),^3^^,^^12–14^ positive urine cultures (PUC; clinical and subclinical; 23.7%-35.7%),^3^^,^^12–19^ and sterile cystitis (3.5-14%).^3^^,^^12–14^^,^^18^

Management of SUB-related complications might require intensive flushing with tetrasodium EDTA, removal or replacement of affected components, antibiosis if infection is present, and supportive care including analgesia.^12^^,^^18^^,^^20^^,^^21^ While the tetrasodium EDTA intensive flushing protocol has achieved promising results for management of mineralization and infected SUB systems, the protocol requires daily, weekly, then monthly veterinary visits, repeated sedation, particularly in fractious cats, and sufficient SUB patency to allow instillation.^20^^,^^21^ Additionally, the associated costs can be considerable, and more frequent routine flushing than advised at the time of SUB implantation might be required after protocol completion.^20^ Clearance of SUB-associated infections, although possible in some cases, is complicated by presumed biofilm formation on implant surfaces, with bacterial colonies embedded within a variably constituted matrix conferring resistance to both the immune system and antibiotics.^22^ Antimicrobial resistance to uropathogens is already reported in cats in the UK,^23^ and prolonged unsuccessful antibiotic courses for symptomatic cases risk the further development of antimicrobial resistance without achieving durable clinical resolution.

SUB explantation for treatment of complications other than SUB-associated gastrointestinal fistula formation remains poorly reported.^24–26^ Restoration of ureteral patency is documented in approximately 80% of cats after SUB placement,^27^ and the SUB might not therefore serve a therapeutic purpose above providing a safety net against potential ureteral re-obstruction. The aim of this study was to describe the clinical characteristics, management strategies, and outcomes of cats undergoing SUB explantation. We hypothesized that SUB explantation would be effective in managing common SUB-related complications and that owners would report a satisfactory outcome for their cat.

## Materials and methods

A retrospective review of medical records was performed for this case series. Ethical approval was obtained from the Royal Veterinary College (RVC) ethical review board (URN SR2023-0148). Patient records of the RVC Queen Mother Hospital for Animals (QMHA) were searched to identify cats that had a SUB managed between October 2015 and August 2023 and cases were reviewed to identify cats that underwent SUB revision or explantation surgery irrespective of the institution of SUB placement. Cats were excluded if SUB explantation was performed at the same hospital visit as SUB placement.

Case data were extracted from the clinical records system including clinical data from the original SUB placement and follow up, including contrast imaging studies, blood work, urinalysis and urine culture, and sensitivity testing. Medical management attempts before explantation were categorized as treatment for sterile cystitis, intensive antibiotic therapy, and/or intensive tetrasodium EDTA SUB flushing without a planned surgical intervention. Clinical findings at the time of SUB explantation, follow up assessments and outcome were also recorded. The reason for SUB explantation was categorized as PUC, sterile cystitis, suspected or confirmed failure of the SUB implant, gastrointestinal fistula, and SUB obstruction based on clinical notes and owner communication logs. SUB obstruction was further subcategorized into implant kink, implant mineralization, or unclear cause.

Where PUC was cited as a reason for SUB explantation, these infections were categorized as “subclinical bacteriuria,” “bacterial cystitis,” or “pyelonephritis” (criteria provided in Supplementary material). Where the designation was not clear from the contemporaneous records, a consensus was reached by 2 board certified small animal internal medicine specialists (SB and JL).

Owners who had agreed to engage in research were contacted and asked “Based on your experience of SUB removal in your cat, would you consider SUB removal again if you were to be in the same situation?” with response options yes, no, or prefer not to answer. If consent was provided, the primary care practitioner was contacted to obtain the complete clinical history.

Data were collated in Microsoft Excel, and descriptive statistics were performed in IBM SPSS statistics (version 29.0.1.0). Normally distributed data are described as mean (± SD) and non-parametric data as median (range). For cats with owner feedback, follow up duration was compared between cats that were alive and dead at the time of contact by Mann–Whitney *U* test. Figures were created in https://BioRender.com.

## Results

Two hundred and forty-five cats with SUBs were managed at the QMHA in the study period. Sixty-two cats underwent revision surgery, SUB explantation, or both during the follow up period. Forty-one cats had only revision surgery performed (*n* = 40), or the SUB was explanted at the same visit as placement (*n* = 1) and were therefore excluded. Twenty-one cats had at least one SUB explanted at a separate visit from initial SUB placement and were included in the analysis, representing 8.6% of all cats managed with a SUB during the study period. These 21 cats comprised 12 neutered females and 9 neutered males. Thirteen cats were non-pedigree. Other breeds represented were Ragdolls (*n* = 2), and one each of Bengal, Bengal cross, British Shorthair, Burmese, Exotic Shorthair, and Siberian. Twenty of the cats had their SUB placed at the QMHA and 1 cat had a SUB placed elsewhere. The mean age at SUB placement was 6.2 years (±3.5 years). Eleven cats had a unilateral SUB and 10 had bilateral SUBs (of which 8 had 2 singles and 2 had a combined system). SUB systems used included the SUB 1.0 (*n* = 18), SUB 2.0 (*n* = 2), and SUB 3.0 (*n* = 1).

In 16 cats, the original cause of ureteral obstruction was ureterolithiasis. In 2 cats, iatrogenic ureteral damage had occurred, with the SUB used to bypass a ureteral repair. In 1 cat, ureteral obstruction was attributed to “cellular material” and in 2 cats the cause of obstruction could not be determined. SUB explantation was performed a median of 1.8 years (0.2-4.1 years) after placement.

**Figure 1:**
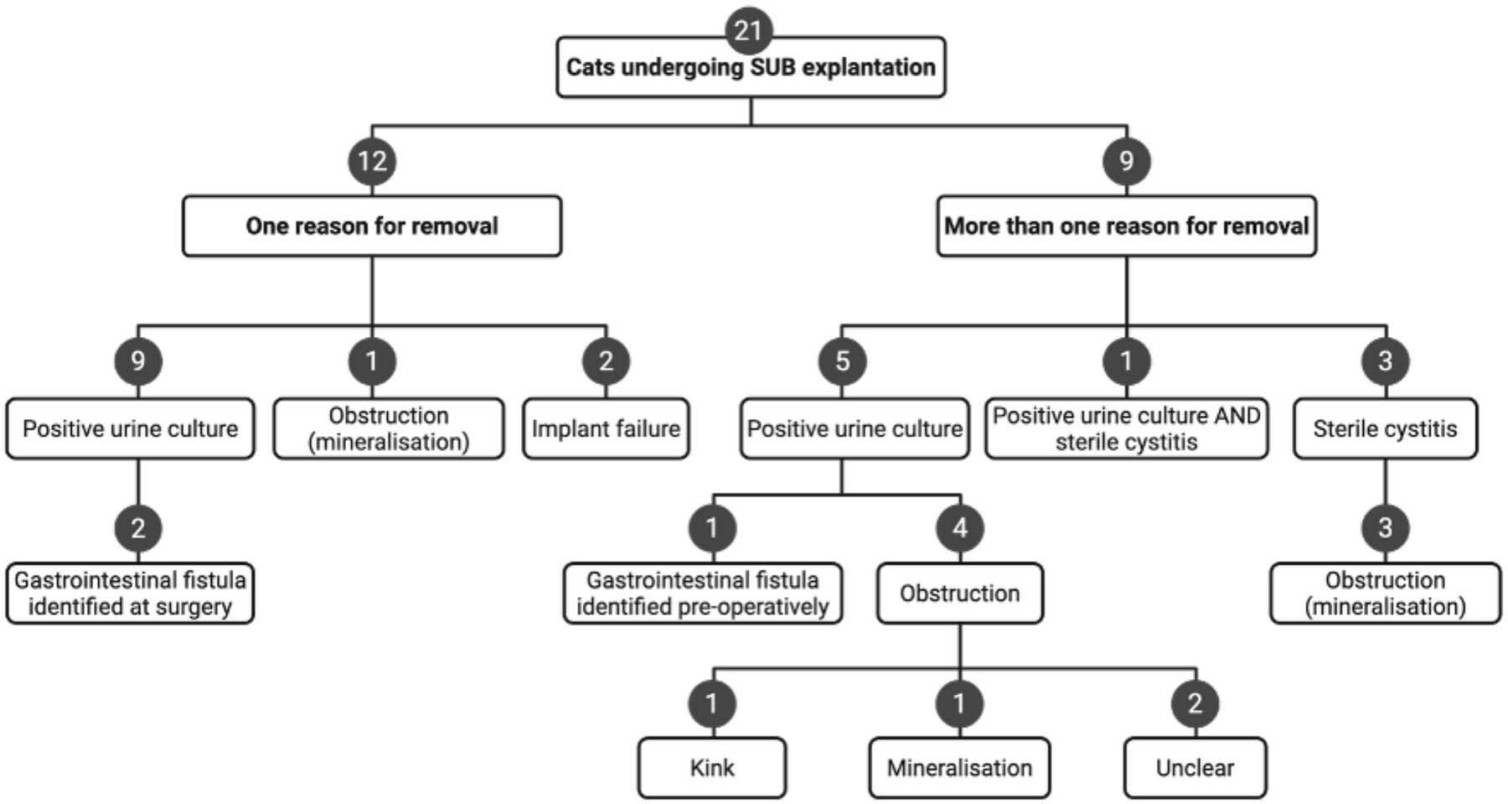
Reasons for subcutaneous ureteral bypass explantation in 21 cats. Numbers denote the number of affected cats.

A single reason for SUB explantation was documented in 12 cats, while 2 reasons were recorded in 9 cats. The most common documented reason for SUB explantation was a PUC (*n* = 15). Obstruction of the SUB in 8 cats contributed to their explantation. Five of these SUBs were mineralized, 1 developed a kink in the nephrostomy catheter very close to the port, and the cause of obstruction was unclear in the remaining 2. Four cats had recurrent sterile cystitis and 1 cat had a gastrointestinal tract fistula identified before surgery. Two cats had a failure or suspected failure of the implant. One of these cats was diagnosed with a uroabdomen and in the second cat a SUB implant fracture was suspected at ultrasound but not confirmed at surgery. Reasons cited for SUB explantation are illustrated in Figure 1.

Of the 15 cats undergoing SUB explantation due to a PUC, the last PUC before surgery yielded a single isolate in 11 cats, 2 isolates in 3 cats and 3 isolates in 1 cat. These 20 isolates comprised *Escherichia coli* (*n* = 13), *Enterococcus faecalis* (*n* = 4), and *Pseudomonas aeruginosa* (*n* = 3). Six isolates were resistant to amoxicillin-clavulanate and 9 were resistant to at least 1 fluoroquinolone. Of the 6 isolates tested against gentamicin and amikacin, none were resistant to gentamicin and 1 isolate was resistant to amikacin. On review of the clinical records, 5 of the PUC cats had signs consistent with subclinical bacteriuria, 5 with bacterial cystitis and 5 cats had signs consistent with pyelonephritis. Of the 5 cats with subclinical bacteriuria, only 1 cat had another reason for SUB explantation (mineralization of the SUB).

### Pre-operative management

Before surgical management, intensive medical management was attempted in 15 cats. Intensive antibiotic therapy was prescribed in 12 cats, with extended courses of amoxicillin-clavulanate in 7 cats, fluoroquinolones in 8 cats, and meropenem, oxytetracycline, and trimethoprim-sulphonamide in a single cat each. In 7 of 12 cats a negative urine culture was obtained while on antibiotics. Ongoing supportive care for sterile cystitis was prescribed in 3 cats and in 1 of these cats an increased frequency of tetrasodium EDTA flushing was additionally initiated to attempt to clear an obstruction. Demineralization protocols were offered for additional cats but were declined by owners.

A total of 31 ureters were assessed for patency by contrast studies including contrast injection into the Shunting Swirl port, pyelography, pyelography via the SUB nephrostomy tube and intravenous urography. These studies occurred either pre- or intra-operatively, and 26 ureters were documented to have regained patency. In those cats without confirmed patency pre-operatively, intraoperative contrast studies were used to guide surgical decision making. The remaining 5 ureters were either not patent, partially obstructed, or the patency could not be determined on the study.

### Surgical management

SUB explantation was a scheduled procedure in 15 of 21 cats. Three additional cats were initially scheduled but required earlier surgery due to clinical deterioration. In 3 cats, SUB explantation had not been planned before the presenting appointment. A total of 17 cats had all implants removed (9 unilateral and 8 bilateral). Two of these cats did not have confirmed ureteral patency by contrast study, one of which had documented partial obstruction and underwent ipsilateral ureteronephrectomy 120 days later. One cat underwent unilateral SUB explantation (due to a gastrointestinal fistula), with the contralateral SUB left in situ although ureteral patency was confirmed. Two cats underwent unilateral SUB explantation with ipsilateral ureteronephrectomy, both of which had pyelonephritis. One of these cats had experienced a renal rupture during attempted medical management, and in the second cat, concern for retroperitoneal fluid was identified on abdominal ultrasound and the owners elected SUB explantation irrespective of ureteral patency. The final cat underwent SUB replacement on the side of ongoing ureteral obstruction, with contralateral SUB explantation; the replacement device was later removed at the primary care practice.

While one gastrointestinal tract fistula was identified pre-operatively, an additional 2 cats were diagnosed with a gastrointestinal fistula intraoperatively. All 3 of these cats had a PUC as one of their reasons for SUB explantation.

### Postoperative findings

Median follow up was 614 days (19-2310 days). Three cats were still alive at time of contact, 810, 2195, and 2310 days after SUB explantation. Eleven cats were lost to follow up at a median of 135 days after SUB explantation (19-1774 days). Seven cats are documented to have died, a median of 627 days (405-2200 days) after SUB explantation. Two of these deaths were unrelated to renal causes, in 1 cat the cause of death was unclear, and in the remaining 4 cats, a renal cause was considered likely. Two of these cats had documented re-obstructions, one had progression of chronic kidney disease, and the final cat had insufficient imaging to determine the cause of azotemia. From the date of initial SUB implantation, cats were followed up for a median of 1282 days (115-3676 days).

Four cats developed re-obstructions at 120, 613, 753, and 1367 days after SUB explantation. All 4 were diagnosed on abdominal ultrasound, either at the QMHA or at the primary care practice. Two cats had originally undergone unilateral SUB placement. One of these cats re-obstructed on the same side as the explanted SUB, and was successfully managed with repeat SUB placement, and 1 experienced a bilateral obstructive episode and was euthanized. The other 2 cats had bilateral SUB explantation. One of these, as previously mentioned, had evidence of partial obstruction at the time of SUB explantation and subsequently underwent ipsilateral ureteronephrectomy. In the remaining cat, the side of re-obstruction could not be determined from the clinical records. Palliative care was provided, and the patient subsequently died. A summary of the reason for SUB implantation, SUB explantation and ureteral re-obstruction event is provided in Table 1.

**Table 1:** Reasons for subcutaneous ureteral bypass (SUB) implantation, explantation, and re-obstruction events in 21 cats with 31 SUBs.

**Cause of initial ureteric obstruction**	**Reason for SUB explantation**	***n***	**Re-obstruction documented (*n*)**
**Urolithiasis**	PUC	13	2
	Obstruction—kink	1	
	Obstruction—mineralization	4	
	Obstruction—unclear cause	2	
	Gastrointestinal fistula	1	
	Sterile cystitis	3	
**Iatrogenic injury**	Implant failure	1	
	PUC	1	1
**Pyonephrosis**	PUC	1	
**Unknown**	Obstruction—mineralization	1	1^a^
	Failure of implant	1	
	Sterile cystitis	1	1^a^

^a^Documents the same cat. Abbreviations: PUC = positive urine culture; SUB = subcutaneous ureteral bypass.

Nine of the 15 cats who had a SUB explanted for a PUC had a negative urine culture postoperatively. In one of these cats, the only negative postoperative urine culture was obtained while on antibiotics. The cat did not have lower urinary tract signs (LUTS) in the clinical records despite recurrent bacteriuria and was followed up until death. Two cats did not have a postoperative urine culture available. Four cats did not have a negative urine culture postoperatively despite 2, 3, 4, and 7 postoperative urine cultures per cat. Two of these cats had negative urine cultures at the time of surgery, but the SUB catheter culture was positive in 1 of these cats. In this second cat a different bacterial species was isolated postoperatively on 4 occasions. The pre-operative bacteria was not isolated again.

No postoperative LUTS were reported in 9 of the 15 cats with a SUB explanted for a PUC. In 3 cats, there was insufficient follow up available to determine the outcome of their PUC, and 3 cats had clinical signs associated with a PUC after SUB explantation. One of these cats had a unilateral replacement at the time of contralateral SUB explantation. This second SUB was subsequently explanted at the primary care veterinarian after recurrent LUTS and documentation of recanalization of the ureter. This cat had originally had the SUB explanted for subclinical bacteriuria. In 1 cat, signs consistent with bacterial cystitis and pyonephrosis occurred over 4 years after SUB explantation for bacterial cystitis, with excellent response to antibiosis. In the final cat, ongoing PUCs and periods of unspecified illness were managed with antibiotics, although the true link was not established. This cat had the SUB removed for pyelonephritis.

### Owner feedback

Owners of 20 cats were contacted for feedback, with 9 owners responding. Eight owners reported that they would choose SUB explantation again under similar circumstances, while one would not. Six of these 9 cats had died a median of 762 days (405-2200 days). after SUB explantation. The other 3 cats were still alive at 810, 2195, and 2310 days after SUB explantation. There was no difference in the length of follow up between cats that were alive and those that had died in this subgroup (*P* = .524).

## Discussion

SUB placement is a frequently employed treatment for ureteral obstructions in cats but complications remain common.^3^^,^^12^^,^^13^^,^^15–20^ This study describes a cohort of 21 cats who underwent SUB explantation to manage one or more device-related complications, with long-term follow-up available in most cases. This represents just under 9% of cats with SUBs managed at our institution during the study period. In the 7 cats who have died during the follow-up period, the median survival was over 600 days after SUB explantation, with one cat living 6 years after surgical explantation of the SUB. Median follow up was just under 3.5 years from SUB placement, far exceeding median survival times for SUB patients in the literature^3^^,^^13^ (albeit with selection of cats who had survived the perioperative period) suggesting that SUB explantation did not negatively impact survival. A PUC was the most common reason for SUB explantation, but other complications including obstruction, sterile cystitis, implant failure, and gastrointestinal fistulae were also managed in this way. After SUB explantation, only 3 cats were documented to have clinical signs of urinary infection, and many achieved a negative urine culture postoperatively, suggesting that SUB explantation can be considered for the treatment of persistent or recurrent urinary tract infections. Ureteral re-obstruction was only documented in a small number of cats and was the cause of death in only half of re-obstructed cats. Owner-reported outcomes were generally favorable in the 9 who answered the questionnaire, with nearly all respondents considering SUB explantation again in the future under similar circumstances.

The complications leading to SUB explantation in this cohort were PUC, SUB obstruction or failure, sterile cystitis, and gastrointestinal fistulas. This is in keeping with prior studies where PUC,^3^^,^^12^^,^^13^^,^^15–19^ obstruction,^3^^,^^12^^,^^13^^,^^18^^,^^20^ and sterile cystitis^3^^,^^12^^,^^13^^,^^16^^,^^18^ are the most common complications and are associated with both welfare and cost considerations. While SUB explantation would be expected to resolve complications such as implant failure, gastrointestinal fistulation, and sterile cystitis, its role in managing SUB obstruction is less straightforward. Given that ureteral patency is reported in over 80% of ureters after SUB placement,^27^ obstruction of the ipsilateral SUB might not always result in clinical signs and therefore SUB explantation might not appear favorable on risk–benefit analysis. Many cats in this study had multiple reasons contributing to explantation of the SUB, with most of the cats with SUB obstruction being in this category. Medical management of SUB mineralization with intensive tetrasodium EDTA flushing can be effective where complete obstruction has not occurred.^20^^,^^21^ Standard SUB flushing at our institution is performed every 3 months, whereas published demineralization protocols advocate for initial daily flushing, decreasing to weekly, biweekly then monthly flushing before returning to a quarterly SUB flush depending on the individual patient’s response.^20^^,^^21^ While the financial impact might be no different to SUB explantation, the requirement for hospital visits, repeated sedation, and prolonged owner commitment might lead owners and veterinarians to consider SUB explantation as an alternative. It is likely that the development of a second complication in the face of a non-functional SUB made the decision to explant easier for both clinician and owner.

Over 70% of cats in this study undergoing SUB explantation had a PUC, but SUB explantation to effectively manage this complication has not previously been reported and was a particular focus of our analysis. Overall, 9 of these 15 cats achieved a negative urine culture postoperatively, and only 3 cats demonstrated signs of a urinary tract infection during follow up. One of these cats had originally undergone unilateral SUB explantation and contralateral SUB replacement. This implant was considered a nidus for ongoing infection and was subsequently removed by the primary care practitioners after documented ureteral patency. Gastrointestinal fistulas were identified in 3 cats with PUC, 2 of which were only detected intraoperatively, and as previously reported, a recurrent PUC in a SUB cat should raise concern for gastrointestinal fistula formation, even in the absence of consistent imaging findings.^25^^,^^26^ Before SUB explantation, many cats were medically managed in the first instance, with 12 cats treated with intensive antibiotic courses with the aim of decolonizing the SUB without surgical intervention. Given the growing drive for judicious antimicrobial use, alternative options to extended or repeated antibiotic courses for management of infected implants are of particular importance.

In 5 cats, the PUC was not associated with clinical signs and would now be considered subclinical bacteriuria (SBU) with the last of these cats managed in July 2019. Evolving knowledge of the urinary microbiome and the potential for a PUC with a non-pathogenic isolate has led to an alteration in management of PUCs in general in cats, but particularly in SUB cats. In one of these 5 cats a second reason for SUB explantation (SUB mineralization) was also present, however, in the remaining 4 cats based on our current management practices, these PUCs would now be managed with benign neglect, if initial attempts to resolve the infection with antibiosis were not successful, and the SUB(s) would not be removed solely due to persistent SBU. This change in cat management practices reflects changes in clinician behavior, experience, SUB system (this study predominantly reporting on SUB 1.0 explantation), and developing conventions in both human and animal medicine.

The cat documented as both sterile cystitis and a PUC reflects the difficulty in distinguishing the clinical impact of bacteriuria in a patient with a SUB implant because the cystostomy tube has the potential to cause bladder irritation and subsequent sterile cystitis.^16^ This case highlights the importance of a detailed urinary history, close assessment of response to therapy and owner training and engagement in their cat’s management.

Of growing recognition in both human and animal research is the emotional toll of renal disease, with 99% of owners of cats with chronic kidney disease reporting feelings of anxiety relating to caring for their cat.^28^ Managing a SUB additionally involves an ongoing financial and time commitment. Owner feedback in this study was largely positive, with most respondents indicating they would consider SUB explantation again if faced with the same situation (specifically if their cat was of the age and health as they were at the time of original decision making). Recognizing the small number of respondents, this information might assist clinicians when counseling owners regarding SUB explantation as a management option, in addition to the long survival times we report. It is important to recognize that the surveyed owners were largely reporting on experience with management of the early SUB system, and further information on outcomes with explantation of the newer SUB systems is needed.

One of the benefits of SUB placement over ureteral surgery for obstructive ureterolithiasis is the presence of the bypass device acting as an ongoing diversion for urine flow if recurrent ureteral obstruction occurs. Therefore, a major concern of SUB explantation in stone-forming cats is the possibility for re-obstruction. Interestingly, we only documented ureteral re-obstruction in 4 cats, one of whom had ongoing partial ureteral obstruction at the time of SUB explantation, despite an average follow up time of nearly 600 days from SUB explantation. The remaining 3 cats, one of whom was successfully managed with repeat SUB placement, re-obstructed at between 613 and 1367 days (1.68-3.74 years), during which time these cats were not requiring SUB flushes—resulting in a cost benefit to the owner and lower patient morbidity. Regular repeat imaging was not performed in all cats, therefore it is likely that some re-obstructions, particularly partial, could have been missed. Where an owner felt that they would not replace a SUB, these findings might not have changed the cat’s management, but this does remain a major limitation of this study.

Our study is limited in many ways by its retrospective nature. Case management was not standardized, as documented improvements to implants and case management led to frequent review of our SUB protocols and might also have contributed to a change in complication rates. The developing knowledge and shift in asymptomatic bacteriuria management in the human field gradually led to a change in the management of subclinical bacteriuria in cats. The drive for improved antibiotic stewardship also affected antibiotic and urine culture choices but equally drove the need for identification of alternative treatments for PUCs. During the study period, the clinicians managing these cats, and the departments primarily responsible for SUB cats also changed.

Full clinical records were not available for all cats with many lost to follow up, limiting outcome conclusions from the entire cohort. As cases were retrospectively reviewed, it is possible that the categorization we applied to cats with a PUC might have been incorrect. This was mitigated by 2 board certified internal medicine specialists reviewing any ambiguous cases. With longer term urine cultures either not reported back to our hospital, or not performed, it is also possible that we have not been able to fully document resolution of infection, or ongoing PUCs after SUB explantation. Many cats did not have postoperative imaging, and it is therefore difficult to confirm the number of cats whose obstruction had truly cleared, but also how many cats suffered a re-obstruction event. While we have reported positive outcomes from SUB explantation, it should be considered that intensive tetraEDTA protocols can resolve mineralization,^20^^,^^21^ and theoretically might assist in the management of PUCs but this has not been fully investigated. While intensive tetraEDTA protocols were offered to some owners in this study, take-up was low, and it is outside of the scope of this study to assess this as a treatment option. However, we acknowledge that this is another option that might allow maintenance of a patent SUB in a cat with a particularly high risk of re-obstruction.

Finally, response bias is always a potential limitation when using a questionnaire as part of a study. With just under half of surveyed owners responding to our questionnaire, our findings are still interpretable; however, we must consider that the remainder of owners might not have been satisfied with the procedure. We did consider whether the owners who responded might have had cats with a longer survival time, or that were still alive, however, this was not the case.

In conclusion, this study reports the largest number of SUB explantations and outcomes in the literature to date with a low number of cats re-obstructing. Many of the cats with a PUC subsequently became urine culture negative and only 3 cats had compatible clinical signs of a urinary tract infection after SUB explantation, although careful distinction between SBU from bacterial cystitis is essential. SUB-associated gastrointestinal fistulas should also be considered in cats with recurrent PUC, irrespective of a negative imaging study. Most of our responding owners would consider SUB explantation again in the future. SUB explantation might represent a viable management option for specific SUB-related complications in carefully selected cases with confirmed ureteral patency, provided owners are prepared for potential future re-obstruction.

## Supplementary Material

Appendix_aalag197

## Off-label antimicrobial declaration

No off-label antimicrobials were used for the purpose of this study. Off-label antimicrobial usage of meropenem as part of clinical treatment unrelated to the study is described.

## Institutional animal care and use committee or other approval declaration

Approved by the Royal Veterinary College (RVC) ethical review board (URN SR2023-0148).

## Abbreviations

LUTS: lower urinary tract signs
PUC: positive urine culture
QMHA: Queen Mother Hospital for Animals
RVC: Royal Veterinary College
SBU: subclinical bacteriuria
SUB: subcutaneous ureteral bypass
