# Supplementary material for: Subcutaneous ureteral bypass explantation in 21 cats (2015-2023): outcomes and owner perceptions
Source: J Vet Intern Med. 2026 Sep 2;40(5):aalag197. doi: 10.1093/jvimsj/aalag197 (PMC13537505; doi:10.1093/jvimsj/aalag197)
Supplement: Supplementary file 1 [file appendix_aalag197.docx]

**Appendix - Supplementary information**

**Materials and methods**

A retrospective review of medical records was performed for this case series. Ethical approval was obtained from the Royal Veterinary College (RVC) ethical review board to contact owners for further follow up (URN SR2023 - 0148). The electronic patient records of the RVC Queen Mother Hospital for Animals (QMHA) were searched to identify cats that had a SUB placed or managed between October 2015 and August 2023. All flagged case records were reviewed by a board-certified internal medicine specialist (SB) to identify cats that underwent SUB revision or explantation surgery after initial device placement. Cats were included if SUB explantation was performed at the QMHA, irrespective of the institution where the SUB had been placed, and clinical records were available. Cats were excluded if SUB explantation was performed at the same hospital visit as SUB placement.

For cats in which SUB explantation was documented at the QMHA at a separate visit from initial placement, case data was extracted from the clinical records system, hospitalization sheets, client communication logs and primary care practitioner clinical records. This included clinical data from the original SUB placement and during follow up, including contrast imaging studies, blood work (and the lab this was performed at), urinalysis and urine culture and sensitivity testing (at any laboratory). Medical management attempts before explantation were categorized as treatment for sterile cystitis, intensive antibiotic therapy, and/or intensive tetrasodium EDTA SUB flushing. Treatment attempts were only reported if the treatment aimed to manage the reported condition, without a planned surgical intervention (for example, antibiotic prescriptions to sterilize urine before surgery were not considered a medical management attempt). Clinical findings at the time of SUB explantation, follow up assessments and outcome were also recorded. The reason for SUB explantation was categorized as PUC, sterile cystitis, suspected or confirmed failure of the SUB implant, gastrointestinal fistula and SUB obstruction based on clinical notes and owner communication logs. SUB obstruction was further subcategorized into implant kink, implant mineralization or unclear cause.

Where PUC was cited as a reason for SUB explantation, these infections were categorized as ‘subclinical bacteriuria’, ‘bacterial cystitis’ or ‘pyelonephritis’. Where the designation was not clear from the contemporaneous records, a consensus was reached by two board certified small animal internal medicine specialists (SB and JL). Subclinical bacteriuria was defined as a PUC in the absence of lower urinary tract signs (LUTS) or systemic signs consistent with pyelonephritis, where LUTS did not respond to appropriate antibiosis or where waxing and waning LUTS were present with variably positive and negative urine culture (i.e. signs were more consistent with sterile cystitis). Bacterial cystitis was diagnosed when a PUC was associated with LUTS, a rapid clinical response to appropriate antibiosis was observed and clinical signs did not recur whilst urine culture remained negative. Finally, a presumptive diagnosis of pyelonephritis was made when a PUC was concurrent with systemic illness, with or without LUTS, with a clinical response to appropriate antibiosis. Additionally, one or more of the following criteria was required: pyrexia, toxic neutrophilia, progressive azotemia, echogenic fluid in the renal pelvis or documented clinical diagnosis of pyelonephritis at the time of treatment.

Hospital consent forms were reviewed to ensure the owners had agreed to engage in research. Consenting owners were contacted by email on a single occasion and provided with an information sheet regarding the study, requesting access to the cat’s up to date clinical records. Owners were additionally asked a single question as yes/no/prefer not to answer. The question was “Based on your experience of SUB removal in your cat, would you consider SUB removal again if you were to be in the same situation?” When email or postal consent was received, the primary care practitioner was contacted to obtain the complete clinical history alongside any blood work and urine culture results performed. Date of death or date of last contact was then confirmed from all available records.

Data were collated in Microsoft Excel, and descriptive statistics were performed in IBM SPSS statistics (version 29.0.1.0). Normally distributed data are described as mean (± standard deviation) and non-parametric data as median (range). Length of follow up in days was calculated from the date of SUB explantation to the date of last contact, death or loss to follow up as appropriate, and time to explantation was calculated in years from the date of initial SUB placement to the date of SUB explantation. In cats with two SUB explantation events the first SUB explantation date was used for follow-up calculations. For cats with owner feedback, the length of follow up was compared between cats that were alive and cats that were deceased at the time of contact by Mann Whitney U test. A *P* value of <.05 was considered statistically significant. Figures were created in https://BioRender.com
